# Improving Efficacy of Endoscopic Diagnosis of Early Gastric Cancer: Gaps to Overcome from the Real-World Practice in Vietnam

**DOI:** 10.1155/2020/7239075

**Published:** 2020-04-26

**Authors:** Duc T. Quach, Quy-Dung D. Ho, Khien V. Vu, Khanh T. Vu, Huy V. Tran, Nhan Q. Le, Nguyen-Phuong N. Tran, Thai H. Duong, Minh C. Dinh, Phuong K. Bo, Xung V. Nguyen, Quy N. Bui, Canh D. Tran, Tien T. Dao, Huong M. Duong

**Affiliations:** ^1^Department of Internal Medicine, University of Medicine and Pharmacy at Ho Chi Minh City, Vietnam; ^2^Department of Gastroenterology, Gia Dinh People's Hospital, Ho Chi Minh City, Vietnam; ^3^Department of Endoscopy, Cho Ray Hospital, Ho Chi Minh City, Vietnam; ^4^Department of Endoscopy, 108 Central Hospital, Hanoi, Vietnam; ^5^Department of Gastroenterology and Hepatology, Bach Mai Hospital, Hanoi, Vietnam; ^6^Gastrointestinal Endoscopy Center, Hue University Hospital, Hue, Vietnam; ^7^Department of Endoscopy, University Medical Center, Ho Chi Minh City, Vietnam; ^8^Department of Endoscopy, Hue Central Hospital, Hue, Vietnam; ^9^Department of Gastroenterology, Thai Nguyen General Hospital, Thai Nguyen, Vietnam; ^10^Department of Gastroenterology, Dong Nai General Hospital, Dong Nai, Vietnam; ^11^Department of Gastroenterology, Can Tho Central General Hospital, Can Tho, Vietnam; ^12^Department of Gastroenterology, Da Nang General Hospital, Da Nang, Vietnam; ^13^Department of Endoscopy and Functional Exploration, National Cancer Hospital, Hanoi, Vietnam; ^14^Department of Diagnostic Imaging, Saint Paul Hospital, Hanoi, Vietnam

## Abstract

**Objective:**

To identify factors associated with increased proportion of early gastric cancer to total detected gastric cancer among patients undergoing diagnostic esophagogastroduodenoscopy.

**Methods:**

A nationwide survey was conducted across 6 central-type and 6 municipal-type Vietnamese hospitals. A questionnaire regarding annual esophagogastroduodenoscopy volume, esophagogastroduodenoscopy preparation, the use of image-enhanced endoscopy, and number of gastric cancer diagnosed in 2018 was sent to each hospital.

**Results:**

The total proportion of early gastric cancer was 4.0% (115/2857). Routine preparation with simethicone and the use of image-enhanced endoscopy were associated with higher proportion of early gastric cancer (OR 1.9, 95% CI: 1.1–3.2, *p* = 0.016; OR 2.7, 95% CI: 1.8–4.0, *p* < 0.001, respectively). Esophagogastroduodenoscopies performed at central-type hospitals were associated with higher proportion of early gastric cancer (OR 1.9, 95% CI: 1.1–3.2, *p* = 0.017). Esophagogastroduodenoscopies performed at hospitals with an annual volume of 30.000–60.000 were associated with higher proportion of early gastric cancer than those performed at hospitals with an annual volume of 10.000-<30.000 (OR 2.7, 95% CI: 1.6–4.8, *p* < 0.001) and with a volume of >60.000–100.000 (OR 2.7, 95% CI: 1.7–4.2, *p* < 0.001). Only four (33.3%) hospitals reported all endoscopic types of early gastric cancer.

**Conclusions:**

The detection of early gastric cancer is still challenging even for endoscopists working in regions with relatively high prevalence. The real-world evidence showed that endoscopic detection of early gastric cancer could potentially improve with simple adjustments of esophagogastroduodenoscopy protocols.

## 1. Introduction

Gastric cancer (GC) is the fifth most common cancer but the third leading cause of death due to cancer worldwide [1]. Endoscopic screening has been recently found to significantly reduce GC mortality in countries with population-based screening program [2, 3]. However, prognosis of the disease is still poor in the rest of the world. A recent survey across 9 countries in the Southeast Asia reported that there were no national screening programs for GC in all countries and the majority of patients were diagnosed in advanced stage with very poor 5-year survival prognosis [4].

One clinically important question is that whether current medical systems could be strengthened for better detection of gastric cancer in early stages. The rationale of this approach is that the miss rate of early GC within 3 years from the indexed esophagogastroduodenoscopy (EGD) was about 18.3% to 22.2% in a high-risk region [5]. In addition, this approach could be quickly implemented compared to the development of national screening programs, which requires important cost-effective analysis and adjustment of government policies.

Vietnam has a population with a high risk of GC but currently has no national screening programs. Vietnam's age-standardized rate of GC is 15.9 per 100.000, which is ranked 10^th^ in the world in 2018, but GC prognosis is very poor with the majority of patients diagnosed in an advanced stage [1, 4]. This study is aimed at assessing the current status of endoscopic diagnosis of early GC and at identifying gaps to overcome so as to improve the detection of early GC.

## 2. Materials and Methods

This study was a survey of the Vietnamese Federation for Digestive Endoscopy (VFDE) in January 2019. A questionnaire regarding esophagogastroduodenoscopy (EGD) preparation, annual EGD volume, modalities of EGD, and number of cases with endoscopic diagnosis of GC in 2018 was sent to nationwide tertiary hospitals which represented for best patients' care in the field of gastrointestinal endoscopy (Table 1). We aimed to assess the current performance of endoscopic detection of GC in early stages in Vietnam at its best so as to develop a platform for future VFDE training activities.

**Figure 1:**
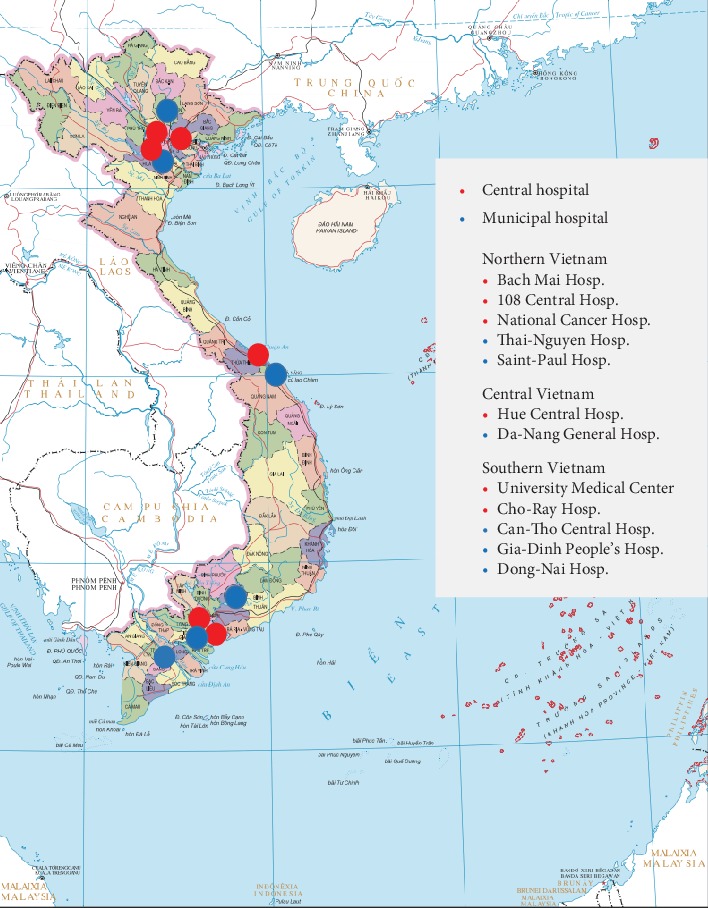
Hospitals which participated in the survey and their locations in the map of Vietnam.

Vietnam has three geographic areas: northern, central, and southern regions. The participating hospitals were selected based on their significant contribution in the field of gastrointestinal endoscopy in their geographic area, which was recognized by VFDE via previous collaborative activities. Totally, there were 12 invited hospitals (6 central and 6 municipal hospitals). In Vietnam, central hospitals are under the direct supervision and support from the Ministry of Health while municipal hospitals are under the direct supervision of the local Department of Health. There were 5 hospitals in southern Vietnam (2 central and 3 municipal hospitals), 2 hospitals in central Vietnam (1 central and 1 municipal hospital), and 5 hospitals in northern Vietnam (3 central and 2 municipal hospitals). Names and locations of the participating hospitals are presented in Figure 1.

The names of participating hospitals were coded to avoid discrimination. Categorical variables are expressed as proportion. Pearson's chi-square test was used to test the difference between proportions of detected early GC between subgroups of patients classified according hospital type, hospital annual EGD volume, simethicone preparation before EGD, use of image-enhanced endoscopy (IEE), and the availability of local surveillance programs. All tests were two-sided and performed at the 5% level of significance. All statistical calculations were performed with SPSS version 20.0 for Windows software (SPSS, Chicago, IL, USA).

## 3. Results

The average annual volume of EGD, preendoscopic preparation, and facilities for EGD at participating hospitals are presented in Table 2. Routine preparation with simethicone and the use of IEE (chromoendoscopy and/or digital IEE) were performed in only 6 (50%) and 4 (33.3%) hospitals, respectively. Totally, the rate of early GC detected in 12 hospitals was 4.0% (115/2857). The endoscopic detection rate of early GC per total GC cases at each hospital ranged from 0 to 7.7% (Table 3).

The factors significantly associated with higher proportion of early GC per total GC cases were EGD performed at central-type hospital (*p* = 0.017), EGD performed at hospitals with an annual volume of 30.000-60.000 (*p* < 0.001), routine preparation with simethicone before EGD (*p* = 0.016), and routine use of IEE during EGD procedure (*p* < 0.001) (Table 4). The number of GCs detected at hospitals which applied local surveillance programs for high-risk patients (*i.e.*, patients with severe gastric atrophy, intestinal metaplasia, and gastric dysplasia) was higher than that detected at hospitals without such programs. However, these programs were not found to associate with significantly higher proportion of early GC (*p* = 0.308).

**Figure 2:**
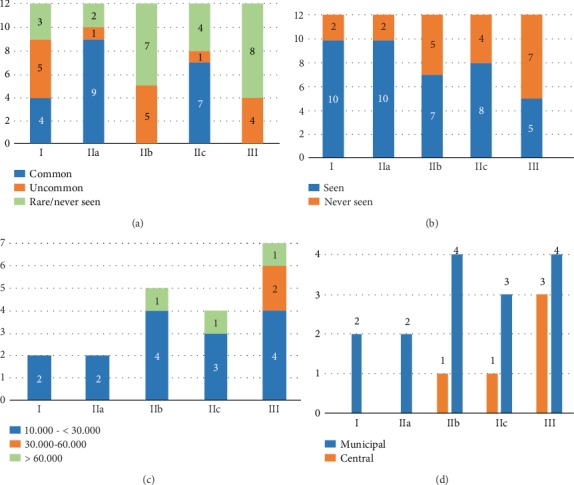
Endoscopic type of early gastric cancer (GC) detected in 12 Vietnamese hospitals in the period of 2008-2018. Common endoscopic types of early GC ever seen (a). Number of hospitals with seen/unseen types of early GC (b). Number of hospitals stratified by annual EGD volume with unseen types of early GC (c). Number of hospitals stratified by hospital type with unseen types of early GC (d).

Regarding the endoscopic type of early GC, only 4 hospitals (2 central and 2 municipal hospitals) reported all types of early GC. The other hospitals, especially municipal hospitals, experienced only some of the endoscopic types of early GC. Type 0-IIa was the most common while types 0-IIb and 0-III were the most uncommon (Figures 2(a) and 2(b)). Less experience with all types of early GC was more popular among hospitals with annual EGD volume < 30.000 (Figure 2(c)) and municipal type (Figure 2(d)). Two municipal hospitals with annual EGD volume < 30.000 reported that there had been no early GC detected during the last 10 years.

## 4. Discussion

GC was the third common cancer and also the third leading cause of death due to cancer in Vietnam [6]. There were 17527 new cases of GC in both sexes diagnosed in Vietnam in 2018 [6]. In this survey, there were 2857 new cases of GC, which accounted for 16.3% of all cases of GC diagnosed in the country in 2018. EGD is popularly used as the first method of investigation for patients with upper gastrointestinal symptoms in Vietnam. It is because Vietnamese patients often concern about GC and request EGD performed. In addition, the cost of EGD in Vietnam is generally acceptable for the majority of Vietnamese. The costs for EGD with local anesthesia and EGD with sedation are 500.000 Dong/21.6 USD and 785.000 Dong/33.8 USD, respectively; and the government insurance covers 226.000 Dong/9.8 USD for both types of procedures. These advantages would make EGD in Vietnam a very potential method of opportunistic screening for GC. It is important to keep in mind the purpose of opportunistic screening whenever diagnostic EGD is indicated. Besides, gaps to overcome so as to improve the efficacy of endoscopic detection of early GC should be investigated.

Regarding EGD routine practice, a similar questionnaire survey in 2016 reported that defoaming agents were used in >90% of Japanese hospitals compared with only 48% of international hospitals outside of Japan [7]. However, IEE was used in a similar way with about 66.7% when GC lesion was suspected. In our study, the rates of routine use of simethicone and selective use of IEE were 50% and 66.7%, respectively (Table 2). The current EGD practice in Vietnamese hospitals was, therefore, almost identical to that in other international hospitals outside of Japan.

We found that EGD performed at central-type hospitals was more likely to detect early GC compared to municipal-type hospitals (OR = 1.918; 95% CI, 1.123–3.277). This could be explained by the different experiences of endoscopists in the two types of hospitals regarding endoscopic findings of early GC and/or some lesions of early GC that had been already detected and referred from primary medical centers. Interestingly, we found that the annual EGD volume of hospital was also significantly associated with the proportion of early GC. The proportion at hospitals with an annual EGD volume of 30.000–60.000 was significantly higher than that at hospitals with an annual EGD volume of 10.000-<30.000 and >60.000–100. 000 (6.4% vs. 2.4% and 2.5%, *p* < 0.001, respectively) (Table 4). On one end, endoscopists at hospitals with a small annual volume could be less experienced with endoscopic findings of early GC. Indeed, we found that most feedback regarding an unseen type of early GC came from small-volume hospitals (Figures 2(c) and 2(d)). Recently, there has been emerging evidence that artificial intelligence could be potentially applied to daily practice as it could process numerous endoscopic images in a very short time with a clinically relevant diagnostic ability [8]. Our survey showed that endoscopic detection of early GC is still very challenging even for endoscopists working in regions with relatively high prevalence. Therefore, artificial intelligence could be potentially useful in real-world practice. On the other hand, we found that the proportion of early GC diagnosed at hospitals with an annual volume of 30.000–60.000 was significantly higher than that at hospitals with an annual volume of >60.000–100.000 (OR = 2.710; 95% CI, 1.718–4.274). The explanation would be not the endoscopists' experience but the overloaded work often experienced at busy Vietnamese gastrointestinal endoscopic centers, which probably related to shorter time of endoscopic examination. The examination time is still a subjective operator-dependent aspect of diagnostic endoscopy. However, there is emerging evidence supporting that among endoscopists with the same level of expertise, the ones who spent more time on EGD examination were more likely to detect GC [9–11]. The recent Asian consensus on the standards of diagnostic upper endoscopy for neoplasia recommended sufficient examination time to increase the detection rate of upper gastrointestinal superficial neoplasm [12]. The result of our survey also suggested the possibility of missing early GC in real-world practice and further supports this recommendation.

Routine preparation with simethicone before EGD and the routine use of IEE during the procedure were found to improve the proportion of early GC with OR = 1.926 (95% CI, 1.128–3.290) and OR = 2.737 (95% CI, 1.858–4.031), respectively. Recent guidelines also recommended these issues with strong evidence from previous randomized control trials [12, 13]. This survey also showed evidence from the real-world practice and supported these recommendations.

In our survey, the proportions of early GC were not significantly different between hospitals with and without local surveillance programs. Currently, there is no national screening program for GC in Vietnam, and some hospitals designed their own surveillance schedules. Recent studies reported that a surveillance interval of every two years would be ideal to detect GC in the early stages [14, 15], but the crucial issue would be the quality of the index EGD endoscopic examination as well as the endoscopist's experience in recognizing EGC [16]. This is an important issue which should be further investigated in future studies.

This study has some limitations. First, as only tertiary hospitals were invited to participate in this study, the whole clinical pictures in Vietnam which would be worse could not be documented. Second, due to the nature of this survey, it was unable to standardize either the preparation with simethicone before the procedure or the practice of IEE in participating hospitals. And this is an important question for future investigations.

In conclusion, the real-world evidence showed that the endoscopic detection of early gastric cancer in Vietnam could potentially improve with simple adjustments of esophagogastroduodenoscopy protocol. Routine preparation with simethicone before EGD and the routine use of IEE during the procedure should be considered. And further training on recognizing endoscopic findings of early GC is important.

## Figures and Tables

**Table 1:** Questionnaire regarding esophagogastroduodenoscopy preparation, modalities, annual volume, and endoscopic diagnosis of gastric cancer.

(1) Your hospital name (2) Your hospital type □ Central hospital □ Province hospital (3) What is the average annual volume of esophagogastroduodenoscopy (EGD) at your hospital? □ 10.000–<30.000 □ 30.000–60.000 □ >60.000–100.000 (4) How often is the preprocedure preparation of EGD with oral simethicone? □ None □ Selective □ Routine (5) How often does the endoscopy unit at your hospital apply chromoendoscopy during EGD? □ None □ Selective □ Routinely (6) How often does the endoscopy unit at your hospital apply digital image-enhanced endoscopy during EGD? □ None □ Selective □ Routine (7) Is magnifying endoscopy available in your hospital? □ Yes □ No (8) Does your hospital have surveillance program for high-risk patients (*i.e.*, patients with gastric dysplasia, intestinal metaplasia and moderate/severe gastric atrophy)? □ Yes □ No (9) Have you documented any cases of early gastric cancer at your hospital over the last 10 years? □ Yes □ No (10) Types of early gastric cancer according to the Japanese classification of gastric carcinoma: kindly number the following type from 1 (for the most common type) to 5 (for the least common type) and 0 for types which have never been seen at your hospital during the last 10 years. □ 0-I □ 0-IIa □ 0-IIb □ 0-IIc □ 0-III (11) Please provide the number of patients with gastric cancer detected by diagnostic EGD in 2018 at your hospital. (12) Please provide the number of patients with early gastric cancer detected by diagnostic EGD in 2018 at your hospital.

**Table 2:** Annual volume, preparation, and facilities of esophagogastroduodenoscopy at participating hospitals.

Hospital characteristics	Number of hospitals, *n* (%)
Hospital type	
Central hospital	6 (50.0)
Municipal hospital	6 (50.0)
Annual EGD volume	
>10.000–30.000	6 (50.0)
30.000–60.000	4 (33.3)
>60.000–100.000	2 (16.7)
EGD preparation with simethicone	
None	2 (16.7)
Selective	4 (33.3)
Routine	6 (50.0)
Chromoendoscopy	
None	2 (16.7)
Selective	9 (75.0)
Routine	1 (8.3)
Digital IEE	
None	1 (8.3)
Selective	7 (58.3)
Routine	4 (33.3)
IEE (either chromoendoscopy or digital IEE)	
None	0
Selective	8 (66.7)
Routine	4 (33.3)
Availability of magnifying endoscopy	
No	5 (41.7)
Yes	7 (58.3)
Surveillance program for high-risk patients	
No	7 (58.3)
Yes	5 (41.7)

EGD: esophagogastroduodenoscopy; IEE: image-enhanced endoscopy.

**Table 3:** Endoscopic detection rates of early gastric cancer across 12 hospitals in 2018.

Hospital ID^∗^	Hospital type	Annual EGD volumes^∗∗^	Number of endoscopists	Simethicone preparation	IEE	ME	Surveillance	EGC (*n*)	GC (*n*)	EGC/GC (%)
1	Municipal	1	7	—	S	—	—	6	78	7.7
2	Central	2	15	R	R	A	Yes	18	245	7.3
3	Central	2	40	R	R	—	Yes	30	447	6.7
4	Municipal	1	7	S	S	—	—	3	48	6.3
5	Central	2	32	R	R	A	Yes	20	348	5.7
6	Municipal	1	14	R	S	—	—	5	96	5.2
7	Central	2	16	S	R	A	—	5	98	5.1
8	Central	3	32	R	S	A	—	13	298	4.3
9	Central	3	28	R	S	A	Yes	13	756	1.7
10	Municipal	1	15	—	S	A	—	2	201	1
11	Municipal	1	8	S	S	A	Yes	0	90	0
12	Municipal	1	10	S	S	—	—	0	152	0

EGD: esophagogastroduodenoscopy; IEE: image-enhanced endoscopy; ME: magnifying endoscopy; R: routine; S: selective; A: available; GC: gastric cancer; EGC: early gastric cancer. ^∗^Names of participating hospitals have been coded to avoid discrimination. ^∗∗^Annual EGD (esophagogastroduodenoscopy) volumes of hospitals were classified as 1 (10.000–<30.000), 2 (30.000–60.000), and 3 (>60.000–100.000) procedures.

**Table 4:** Factors associated with higher proportion of detected early gastric cancer.

	EGC/GC (%) (*n*/*N*)	*p*	Odd ratios (95% confidence interval)
Hospital type			
Municipal	2.4 (16/665)	0.017	1
Central	4.5 (99/2192)		1.918 (1.123–3.277)
Hospital volume (EGD procedures/year)			
10.000–< 30.000	2.4 (16/665)	—	1
30.000–60.000	6.4 (73/1138)	<0.001	2.780 (1.604–4.818)
>60.000–100.000	2.5 (26/1054)	0.936	1.025 (0.546–1.927)
Preparation with simethicone before EGD			
None/selective	2.4 (16/667)	0.016	1
Routine	4.5 (99/2190)		1.926 (1.128–3.290)
Image-enhanced endoscopy			
Selective	2.4 (42/1719)	<0.001	1
Routine	6.4 (73/1138)		2.737 (1.858–4.031)
Surveillance for high-risk subjects			
No	3.5 (34/971)	0.308	1
Yes	4.3 (81/1886)		1.237 (0.822–1.860)

EGC: early gastric cancer; GC: gastric cancer; EGD: esophagogastroduodenoscopy.

## Data Availability

The data used to support the findings of this study are available from the corresponding author upon request.
